# Association between obesity and sickness in the past two weeks among middle-aged and elderly women: A cross-sectional study in Southern China

**DOI:** 10.1371/journal.pone.0203034

**Published:** 2018-08-28

**Authors:** Li-Ying Fu, Xiao-Xiao Wang, Xiao Wu, Bo Li, Ling-Ling Huang, Bing-Bing Li, Qing-Feng Du, Pei-Xi Wang

**Affiliations:** 1 Institute of Chronic Disease Risks Assessment, School of Nursing and Health, Henan University, Kaifeng, Henan Province, China; 2 General Practice Center, Nanhai Hospital, Southern Medical University, Guangzhou, Guangdong Province, China; 3 Nursing Department, Tongji Hospital, Tongji Medical college, Huazhong University of Science and Technology, Wuhan, Hubei Province, China; 4 Department of Preventive Medicine, School of Public Health, Guangzhou Medical University, Guangzhou, Guangdong Province, China; Hong Kong Polytechnic University, HONG KONG

## Abstract

**Objectives:**

Sickness situation in the past two weeks, an indicator of health service needs, is an increasing major health concern. However, data on the relationship between obesity and two-week morbidity in the female population, particularly in middle-aged and elderly women, is sparse. The present study aimed to examine the association between obesity and two-week morbidity among middle-aged and elderly women in Southern China, and to explore the independent contributions of socio-demographic variables, health-related factors, and obesity to two-week morbidity.

**Methods:**

In total, 2364 middle-aged and elderly women were included in this cross-sectional, community-based survey. Obesity was assessed using body mass index (BMI). The outcome variable was sickness situation over the past two weeks (two-week morbidity). Clustered logistic regression was applied to analyze the independent contribution of obesity to two-week morbidity.

**Results:**

Approximately 14.6% of participants experienced sickness in the past two weeks. Obesity (odds ratio [OR] = 1.47, 95% confidence interval [CI] = 1.02–2.12) was significantly associated with two-week morbidity and its independent contribution accounted for 3.7%, lower than that of socio-demographic variables (73.7%) and health-related factors(22.6%).

**Conclusions:**

Some degree of correlation was observed between obesity and two-week morbidity among middle-aged and elderly women in Southern China, which can be used as a reference for health-related decision-making.

## Introduction

With the rapid increase in global population and the changes in social structure and lifestyle, the prevalence of obesity has rapidly increased over the past four decades worldwide, reaching 18% in men and surpassing 21% in women by 2025, and this has become a major concern [1,2]. As a global epidemic, the number of obese adults exceeded that of underweight adults, particularly in China, which has been on top of the list worldwide. Currently, women account for more than half of the total obese population [3]. Health risks increase with exce**s**s weight [4], leading to substantial morbidity and disability, increased work absenteeism due to sickness [5–8], impaired quality of life [9], increased rates of hospitalizations [10], and increased risk for mortality [11–13]. In addition, obesity does not only cause a heavy burden on our health care system, but it has a significant impact on medical costs [14–16]. Accordingly, future studies on obesity and its effects must be performed.

Two-week morbidity is defined as the proportion of individuals who get sick in the past two weeks [17], a primary indicator of overall health status and health service needs of residents in China [18], which significantly identifies the health service content and mode of operation. Numerous epidemiological studies on two-week morbidity have been conducted to identify health service needs and to provide references for health decision-making. The prevalence rate of sickness in the last two weeks varied between 4.9% and 40.6% in the community residents, whereas it ranged from 5.4% to 38.9% among women [18,19]. The wide variations in the two-week morbidity rate in previous studies may be due to the different study designs and characteristics of participants. Generally, the two-week morbidity rate varied by age [19–21]. Elderly individuals have a higher two-week morbidity, with no exception women in rural areas [22]. In addition, two-week morbidity may denote the first signs of emerging health problems and lead to confinement in bed and absence from work for a significant number of days in China [21]. To reduce the two-week morbidity and its associated adverse outcomes in middle-aged and elderly women, the risk factors of two-week morbidity must be identified and understood.

However, referring to the effect of the risk factors on sickness in the last two weeks, numerous studies have paid more attention to the demographic data of the participants, while studies on health-related factors, particularly obesity, have been much less studied. More interestingly, a population-based study in Hangzhou showed that obese individuals were more likely to be sick in the past two weeks than normal-weight individuals [18], but what is at odds with this finding is that obesity was not significantly associated with the two-week illness [19,23]. Unfortunately, the controversy was failed to be deeply studied in China. In addition, the effect of obesity on two-week morbidity was indirectly confirmed by assessing the impact of obesity on short-term illness overseas [5–8].Based on an in-depth literature review, Body mass index (BMI) has been widely and universally used to measure obesity in studies on two-week morbidity. Therefore, assessment on the association between obesity and two-week morbidity based on the Chinese standard criteria for obesity is important.

Having been inspired by the aforementioned considerations, the present study aimed to examine the association between obesity and two-week morbidity using BMI as a validation index. Furthermore, the independent contributions of socio-demographic variables, health-related factors, and obesity to two-week morbidity in middle-aged and elderly women in southern China were examined.

## Materials and methods

### Study design and participants

This study was based on a cross-sectional community health survey in Guangdong province, Southern China, 2017. The survey samples were selected using a multistage and stratified random sampling method. The primary sampling units were street communities, second-stage sampling units were communities and the stratification was according to the economic level. A total of 2643 women aged 45 and older were approached, 192(7.3%) of them refused participation or did not respond, 177 of them provided questionnaires with incomplete data. Finally, the total number of women included in the analysis was 2364.

### Procedures

Data were collected via face-to-face household interviews by trained personnel. An interview group comprising a medical student (Guangzhou Medical University) and a local healthcare staff member (community health service center) collected research data using standard-structured questionnaires. The interviewers received the same training prior to the survey to standardize data collection and recording procedures. In addition, each group was accompanied by a supervisor to ensure that the interviews were completed accurately. On average, each participant completed the interview for approximately 30 minutes.

### Ethics statement

This study was approved by the Ethics Committee of Guangzhou Medical University. Written informed consent was obtained from each study participant prior to investigation.

### Measurements

We first conducted a literature search to identify the potential factors associated with two-week morbidity. The variables found through this search were classified into three: socio-demographic characteristics (age, marital status, education level, etc.), health-related factors (current smoking, alcohol drinking, regular exercise, etc.), and obesity.

#### Socio-demographic characteristics

Marital status was dichotomized into married and single, “married” as ‘‘married” while ‘‘divorced/widowed/single” as ‘‘single”. Educational level was categorized into primary school or lower, middle school, high school or higher. Employment status was divided into three groups: employed, retired, and unemployed.

#### Health-related variables

Current smoking status was defined as smoking one or more cigarettes per day for at least 6 months. Alcohol drinking was defined was defined as drinking alcohol for participants who reported consuming alcohol an average of more than once a week within the last year. Regular exercise was defined as participating in moderate or intensive activity lasting no less than 30 minutes ≥ 3 times per week, except for routine office and house work. Time of sitting was defined based on the response to the question “how long do you sit on an average day?”

#### BMI

Weight was determined to the nearest 0.1 kg on a portable batheroom scales, and height was measured to the nearest 0.1 cm with a tape measure by a trained investigator with standard methods. According to the Chinese BMI reference [24], BMI was calculated according to the standard formula, i.e. weight in kilograms divided by squared height in meters, and all study participants were categorized as underweight (<18.5 kg/m^2^), normal weight (18.5–23.9 kg/m^2^), overweight (24.0–27.9 kg/m^2^), and obese (≥ 28.0 kg/m^2^).

#### Dependent variables

The concept of sickness in the past two weeks was defined if the respondents had any of the following three circumstances in the prior two weeks when interviewed: (1) conscious feeling of discomfort, seeking diagnosis and treatment; (2) conscious feeling of discomfort, not seeking diagnosis and treatment but choosing self-treatment; (3) conscious feeling of discomfort, not seeking diagnosis and treatment and choosing self-treatment but being absent from work and school or lying in bed for at least one day because of discomfort [22,25].

The “sickness” defined by the health service research is considered from the perspective of resident’s health service needs. It is not the “sickness” in the strict sense and mainly based on the subjective feelings of the respondents and the objective judgment of the trained investigators [26]. Thereafter, the two-week morbidity was defined as “number of respondents who had sickness in the past two weeks divided by total number of respondents × 100%”[19,23,25,26].

### Data analysis

Mean and standard deviations (SD) were presented for continuous variables, while frequency and percentage were used for categorical variables. The main dichotomous outcome variable was two-week morbidity. To assess the differences in the distributions based on continuous or categorical variables, t-test or chi-square test was used accordingly. Logistic regression was employed to calculate the odds ratios (ORs) and 95% confidence intervals (95% CIs). Continuous variables (BMI) were standardized to make the data comparable.

Subsequently, clustered logistic regression was used to explore the impacts of socio-demographic characteristics, health-related factors, and obesity (three clusters) on sickness in the last two weeks[27,28]. Multidirectional associations may exist among the three clusters and the dependent variable. To be specific, cluster 1 may affect cluster 2, cluster 3, and the outcome variable. Likewise, cluster 2 may affect cluster 3 and the dependent variable, whereas cluster 3 may affect the outcome variable. Consequently, the simultaneous consideration of variables from the clusters in a free multiple regression model (i.e., a free forward stepwise logistic regression model) might cause confounded inference. Thus, we adopted clustered logistic regression[27], which may facilitate a more realistic assessment of the relative contribution of the factors to sickness in the last two weeks. The regression model was determined in three steps: (1) A forward stepwise regression of sickness in the past two weeks for the cluster 1 variables; (2) A forward stepwise regression for the cluster 2 variables with the equation derived from step 1 as a fixed part of the new regression model; (3) A forward stepwise regression for the cluster 3 variables with the equation derived from step 2 as a fixed part of the new regression model. The variables for the inclusion and exclusion criteria for the stepwise regression models were P values of 0.05 and 0.10, respectively.

The independent effect of each cluster was assessed using the corresponding R^2^ value. The independent contribution share of each cluster was calculated as individual R^2^ change/total R^2^ change in the final model × 100%. The R^2^ in logistic regression models was the Nagelkerke “pseudo” R^2^, similar to the classical R^2^ in linear regression models for data interpretation [28].

All analyses were carried out using the Statistical Package for the Social Sciences (SPSS) version 13.0 (SPSS Inc., Chicago, IL, USA). Two-tailed P values less than 0.05 were considered statistically significant.

## Results

### Participant characteristics

Descriptive statistics on the study variables are presented in Table 1. Among 2364 middle-aged and elderly women (aged 45 years and older), with an average age of 60.2 ± 11.1 years, 2010 (85.0%) were married, and 354 (15.0%) were single. Majority of the participants received an education of primary school or lower (52.4%), they had medical insurance (89.8%). With regard to health-related factors, most did not smoke (99.1%), drink (94.7%), exercised regularly (55.9%) and sat for more than 6 hours per day on average (59.4%). The average BMI was 23.7 ± 3.8 kg/m^2^. Meanwhile, we divided the participants according to the presence of sickness in the past two weeks. The results of the chi-square test and t-test showed that the variables between the two groups differed significantly in terms of age, marital status, education level, employment status, regular exercise, and BMI (*P* < 0.05) (Table 1). In addition, approximately 14.6% of women experienced illness in the last two weeks before the survey.

**Table 1:** Participant characteristics (N = 2364 middle-aged and elderly women) according to dichotomized two-week morbidity in 2017.

Variables	Two-week morbidity		Total (n = 2364)	*P*
	Yes (n = 345)	No (n = 2019)		
**Cluster 1(socio-demographic)**				
Age, years (m, SD)	64.7± 11.4	59.4 ± 10.8	60.2 ± 11.1	<0.001***
Age, years (n, %)				<0.001***
45–59	110 (9.0)	1112 (91.0)	1222 (51.7)	
60–74	163 (18.9)	699 (81.1)	862 (36.5)	
≥75	72 (25.7)	208 (74.3)	280 (11.8)	
Marital status (n, %)				<0.001***
Married	253 (12.6)	1757 (87.4)	2010 (85.0)	
Single	92 (26.0)	262 (74.0)	354 (15.0)	
Education level (n, %)				<0.001***
Primary school or lower	257 (20.7)	982 (79.3)	1239 (52.4)	
Middle school	55 (8.4)	600 (91.6)	655 (27.7)	
High school or above	33 (7.0)	437 (93.0)	470 (19.9)	
Employment status (n, %)				<0.001***
Employed	53 (8.4)	575 (91.6)	628 (26.6)	
Retired	105 (12.8)	718 (87.2)	823 (34.8)	
Unemployed	187 (20.5)	726 (79.5)	913 (38.6)	
Medical insurance (n, %)				0.337
Yes	315 (14.8)	1807 (85.2)	2122 (89.8)	
No	30 (12.4)	212 (87.6)	242 (10.2)	
**Cluster 2 (health-related)**				
Current smoking (n, %)				0.355
Yes	5 (22.7)	17 (77.3)	22 (0.9)	
No	340 (14.5)	2002 (85.5)	2342 (99.1)	
Alcohol drinking (n, %)				
Yes	16 (12.7)	110 (87.3)	126 (5.3)	0.605
No	329 (14.7)	1909 (85.3)	2238 (94.7)	
Regular exercise (n, %)				<0.001***
Yes	135 (10.2)	1187 (89.8)	1322 (55.9)	
No	210 (20.2)	832 (79.8)	1042 (44.1)	
Time of sitting, hours (n, %)				
<6	205 (14.6)	1199 (85.4)	1404 (59.4)	1.000
≥6	140 (14.6)	820 (85.4)	960 (40.6)	
**Cluster 3 (BMI)**				
BMI (m, SD)	24.6 ± 4.3	23.6 ± 3.7	23.7 ± 3.8	<0.001***
BMI, kg/m^2^(n, %)				0.002**
Normal weight (<18.5)	170 (13.7)	1075 (86.3)	1245 (52.7)	
Underweight (18.5–23.9)	11 (10.9)	90 (89.1)	101(4.3)	
Overweight (24.0–27.9)	110 (14.1)	669 (85.9)	779 (33.0)	
Obese (≥28.0)	54 (22.6)	185 (77.4)	239 (10.1)	

Note: Data presented are mean ± SD or n (%). Single: unmarried, divorced or widowed. BMI: body mass index.

* *P* < 0.05

** *P*< 0.01

*** *P* < 0.001.

### Two-week morbidity and BMI

**Fig 1:**
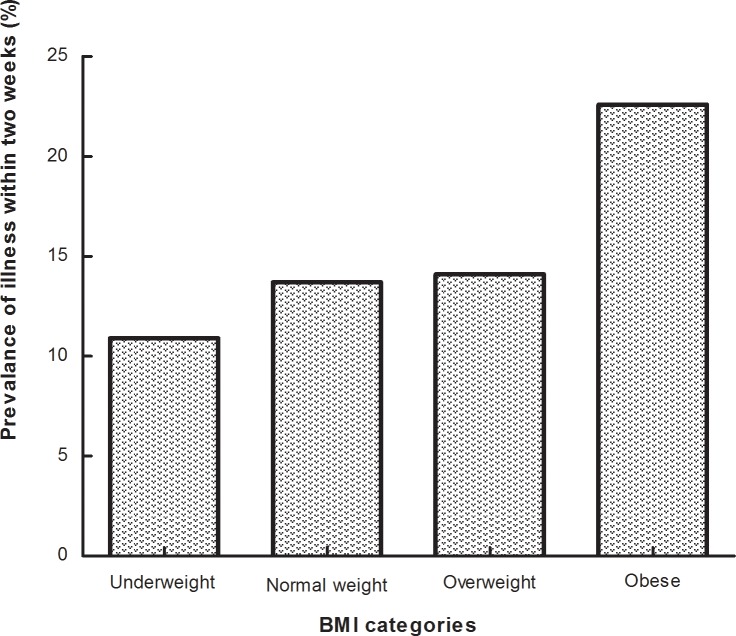
Prevalence of sickness in the past two weeks in different BMI categories.

The two-week morbidity rate was highest in obese women with reference to the normal weight group (22.6% vs 13.7%) (Table 1 and Fig 1). In the univariate analysis, Compare with the normal weight group, obese participants had a near two times risk of two-week morbidity (crude OR = 1.85, 95% CI = 1.31–2.60, *P* < 0.001), and there were no difference in the underweight and overweight group.(Table 2).

**Table 2:** Association of obesity with sickness in the past two weeks^a^.

Predictor variables	OR	95% CI	*P*
BMI			0.003**
Normal weight (<18.5)	Reference		
Underweight (18.5–23.9)	0.77	0.41–1.48	0.435
Overweight (24.0–27.9)	1.04	0.80–1.35	0.768
Obese (≥28.0)	1.85	1.31–2.60	<0.001***

Note: BMI: body mass index, OR = odds ratio, CI = confidence interval.

^a^ BMI was included as predictor variables for sickness in the past two weeks in univariate logistic regression model without adjusting for other variables.

* *P* < 0.05

** *P*< 0.01

*** *P* < 0.001.

### Determinants of two-week morbidity

**Fig 2:**
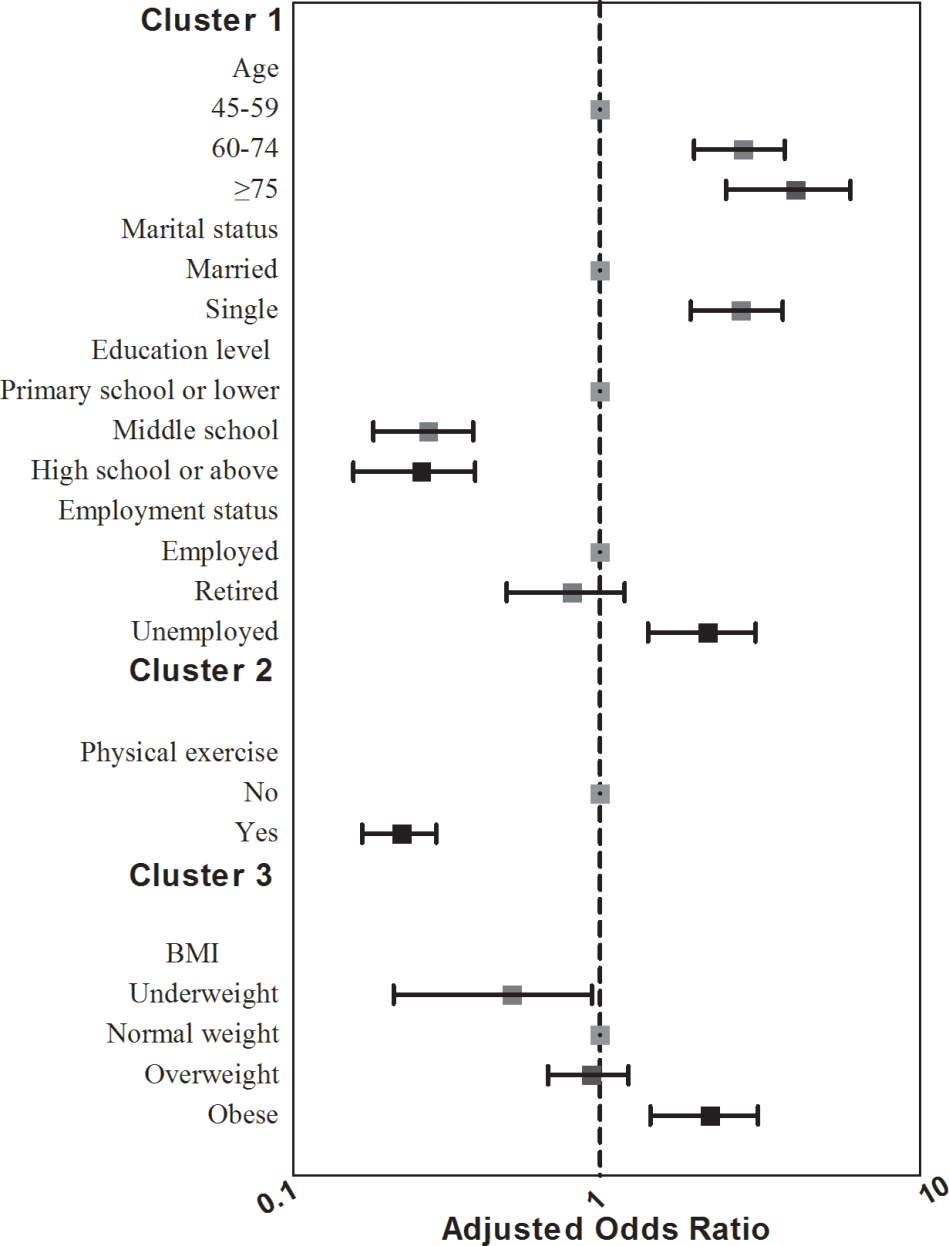
Adjusted odds ratios for two-week morbidity with 95% confidence intervals by cluster 1, cluster 2 and cluster 3.

In the clustered logistic regression analysis, the variables included in the final progressive cluster model of logistic regression are shown in Table 3. The ORs were adjusted for other variables and are illustrated in Fig 2. Among the socio-demographic variables (cluster 1), age, marital status, education level, and employment status were significantly associated with sickness in the past two weeks. Two elderly groups (60–74, ≥75 years) were almost 2-fold more likely to be sick than the reference group (45–59 years), respectively (*P* < 0.05). Women who were single and those who went to middle or high school or higher were at higher risk of becoming sick in the last two weeks (*P* < 0.001). Unemployed women were almost 1.5 times more likely to be sick in the past two weeks (*P* < 0.05).The independent contributions of socio-demographic variables were 73.7%. In cluster 2 (health-related factors), only regular exercise was associated with the decreased risk of illness in the last two weeks (*P* < 0.001). The independent contributions of the second cluster in middle-aged and elderly women were 22.6%. In the third cluster, compared with the normal-weight group, obese participants were nearly 1.5 times at risk of two-week morbidity (OR = 1.47, 95% CI = 1.02–2.12, *P* = 0.039), and no difference was observed in the underweight and overweight groups. The independent contributions on the risk of being sick in the last two weeks were 3.7%.

**Table 3:** Cluster logistic regression models explaining sickness in the past two weeks by socio-demographic characteristics (cluster 1), health-related factors (cluster 2) and obesity (cluster 3).

Predictor variables	OR (95% CI)	*P*	Nagelkerke^a^ R^2^	Independent contribution(%)^b^
**Cluster 1**				
Age				
45–59	Reference			
60–74	1.70 (1.25–2.31)	0.001**		
≥75	2.03 (1.33–3.10)	0.001**		
Marital status				
Married	Reference			
Single	1.68 (1.23–2.30)	0.001**		
Education level				
Primary school or lower	Reference			
Middle school	0.49 (0.35–0.69)	<0.001***		
High school or above	0.47 (0.31–0.71)	<0.001***		
Employment status				
Employed	Reference			
Retired	0.85 (0.57–1.27)	0.436		
Unemployed	1.46 (1.01–2.10)	0.045*		
Total			0.101	73.7
**Cluster 2**				
Physical exercise				
No	Reference			
Yes	0.45 (0.35–0.58)	<0.001***		
Total			0.132	22.6
**Cluster 3**				
BMI				
Underweight (<18.5)	0.61 (0.31–1.20)	0.150		
Normal weight (18.5–23.9)	Reference			
Overweight (24.0–27.9)	0.94 (0.72–1.24)	0.671		
Obese (≥28.0)	1.47 (1.02–2.12)	0.039*		
Total			0.137	3.7

BMI: body mass index, OR = odds ratio, CI = confidence interval.

^a^ Nagelkerke R^2^ is the variance of the dependent variable (two-week morbidity) explained by all independent variables included in the regression model.

^b^ The independent contribution of each cluster of predictors to the variation in sickness in the past two weeks was calculated as individual corresponding R^2^ change/total R^2^ change in the final model×100%.

* *P* < 0.05

** *P*< 0.01

*** *P* < 0.001.

## Discussion

### Main findings

To the best of our knowledge, this was the first study showing the association between obesity and two-week morbidity in middle-aged and elderly women in Southern China. Approximately 14.6% of women aged 45 years and older got sick in the past two weeks. Obesity was significantly associated with two-week morbidity after adjusting for socio-demographic and health-related factors. However, the independent contribution of obesity to two-week morbidity was minimal. These findings suggested that some degree of correlation was observed between obesity and two-week morbidity among middle-aged and elderly women.

### Comparison with previous studies

With the samples drawn in the current study, the morbidity rate was 14.6% among the present study population in the past two weeks, which indicated the poor health status among the middle-aged and elderly women. Wen and colleagues have revealed that the two-week morbidity rate was 13.4% among women in rural areas[22],which was in accordance with the result of our study. However, other studies have reported different two-week morbidity rates. For instance, a study conducted in a western Chinese city has shown that the two-week prevalence rate in the female residents of rural areas was 8.0%[29]. Zhu and colleagues have revealed that the two-week morbidity rate was only 3.42% among rural-urban migrate workers[30]. The differences might be partially explained by the specific characteristics of the study participants, methodology, and region disparity. In brief, the risk factors associated with the high prevalence of sickness in the last two weeks must be identified to initiate preventive measures and health care.

Our study showed that obesity was significantly associated with two-week morbidity among middle-aged and elderly women, even after adjusting for socio-demographic and health-related factors. Obese women had nearly 1.5 times higher risk odds of being sick in the last two weeks than women with normal weight. Our results correspond to the findings of studies that had examined BMI and two-week morbidity and showed that obesity has a negative effect on community residents [18,20]. Lin et al. have shown that the risk of two-week morbidity due to overweight and obesity was 1.64 and 3.38 times than that of normal or thinner individuals living in Chinese communities, respectively [20]. However, a community-based research with relatively small sample sizes have revealed that obesity was not significantly associated with two-week morbidity [23]. The difference in the findings of these studies conducted in China may result from the different region and characteristics of the participants. In addition, this association between obesity and two-week morbidity was also indirectly reflected by assessing the impact of obesity on short-term illness overseas. Khongsdier and colleagues have verified that the risk of becoming sick during the last four weeks was higher in participants with a high body-fat mass index than the normal group [31]. Another cohort study found that stable obesity increased the risk for short sickness absence among women [6], as shown in previous studies [5,7,8]. Unfortunately, most of previous study had a small sample size and did not account for necessary health-related confounders [18,31], such as regular exercise and sitting time [20,23]. The important findings in our study were that the relationship between obesity and two-week morbidity remained significant after controlling for socio-demographic and health-related factors among middle-aged and elderly women. The association may be caused by the tendency that obese participants are more prone to suffer from obesity-related chronic diseases, which results in an increased two-week prevalence rate. Jing and colleagues have elucidated that obesity was a risk factor associated with fatigue [32], which might also affect health status and lead to sickness in the past two weeks. More interestingly, certain studies have also observed a U-shaped relationship between BMI and mortality and other adverse outcomes, with the highest mortality risk observed in the underweight group [33]. Conversely, in our study, only obesity was significantly associated with two weeks of illness, and underweight individuals had the lowest two-week morbidity among middle-aged and elderly women. The lean body mass of the underweight group may partly explain this difference. In summary, the associations between obesity and two-week morbidity are complex and not fully understood. Thus, more studies must be conducted in the future.

The independent contribution of the third cluster to two-week morbidity was minimal. Individuals are more likely to seek treatment or present with sickness in the last two weeks due to individual characteristics or lifestyle habits rather than obesity. Two-week morbidity is a sensitive index reflecting the recent health status of residents and recent health service requirements. Health risks increased with excess weight in a prospective study by Roos and colleagues [6]. It might be partially explained by the fact that two weeks is an extremely short period of time for evaluating the impact of BMI on recent poor health status and two-week morbidity. Future studies must consider a longer follow-up period for assessing the impact on recent health service needs. Of the social demographic factors, age, marital status, education level, and employment status have been associated with two-week morbidity, as shown in previous studies [19,22]. In general, with aging, individuals become less energetic, with deteriorating physiological functions and increased risk for short-term diseases. Single status, which is a risk factor of social support from family members, could also increase the likelihood of two-week morbidity [34]. A higher education level may indicate a higher quality of life [35] and lower number of reported health problems, which were obviously associated with two-week morbidity [36]. As for health-related factors, only regular exercise was significantly associated with two-week morbidity. The positive effects of physical activity, such as improving physical conditions [34]and enhancing psychological well-being [37], which have been emphasized in older adults[38] are possible reasons. However, one literature has shown no significant difference between two-week morbidity and physical exercise among residents aged 65 years [39]. Thus, a better understanding of health-related lifestyle factors may help in the early development and utilization of strategies for health promotion in elderly individuals.

The present study had several limitations. First, the data in our analyses were based on self-reports, which could lead to biases or could have inaccuracies, although physical examinations were conducted to record their height and body weight. Second, the evaluation of obesity with BMI is cumbersome. BMI does not differentiate body fat distribution or body fat from lean mass, which is associated with increased two-week morbidity. However, BMI is widely and universally used, and it is easily accessible for general practice and fairly reliable in terms of body fat percentage and body fat mass in both developed and developing countries [31]. Third, income was not included in this study considering that income reporting is a sensitive issue and not reliable in China, but we have data on employment status which may partly reflect the relative income status. In addition, we have no data on the residence which is another factor associated with morbidity. Nevertheless, it is known that the Pearl River Delta Region of Guangdong province, our survey field, has basically completed the urbanization now. Almost all the residents are managed within the jurisdiction of the community currently. Finally, considering the cross-sectional study design, the observed associations could not be assumed as causal relationships. Further in-depth studies with longitudinal follow-up data are warranted to explore the cause and effect relationship.

## Conclusions

The two-week morbidity in middle-age and older women was high. Obesity was significantly associated with two-week morbidity among middle-aged women. However, the independent contribution of obesity on two-week morbidity was minimal. These findings suggest that the importance of obesity on the health condition of middle-aged and elderly women in the past two weeks should be considered appropriately. Obesity, as an effective indicator, should be considered reasonably and in a timely manner to help health practitioners provide counseling on the consequences of obesity and help policy makers direct public health messages.

## Supporting information

S1 Dataset Association between obesity and sickness in the past two weeks among middle-aged and elderly women. (XLSX)

S1 Questionnaire Community diagnostic questionnaire. (PDF)
