# Supplementary material for: Association between obesity and sickness in the past two weeks among middle-aged and elderly women: A cross-sectional study in Southern China
Source: PLoS One. 2018 Aug 28;13(8):e0203034. doi: 10.1371/journal.pone.0203034 (PMC6112645; doi:10.1371/journal.pone.0203034)
Supplement: S1 Questionnaire — (PDF) [file pone.0203034.s002.pdf]

社区诊断调查表

户主姓名：______________ 电话号码：__________________________

现住址：______________________________ 住户代码：____________

身份证号码：_ _ _ _ _ _ _ _ _ _ _ _ _ _ _ _ _ _

成员 02 姓名________ 身份证号码：_ _ _ _ _ _ _ _ _ _ _ _ _ _ _ _ _ _
成员 03 姓名________ 身份证号码：_ _ _ _ _ _ _ _ _ _ _ _ _ _ _ _ _ _
成员 04 姓名________ 身份证号码：_ _ _ _ _ _ _ _ _ _ _ _ _ _ _ _ _ _
成员 05 姓名________ 身份证号码：_ _ _ _ _ _ _ _ _ _ _ _ _ _ _ _ _ _
成员 06 姓名________ 身份证号码：_ _ _ _ _ _ _ _ _ _ _ _ _ _ _ _ _ _

已介绍本次社区卫生诊断目的，取得了被调查居民户的知情同意☐ 签名：____________
调查日期：_________年_______月________日

一级质控员（签名）：__________ 二级质控员（签名）：__________

核实日期： 年 月 日 核实日期： 年 月 日

您好！我们是社区诊断调查的调查员。本次调查内容基于国家卫生服务调查根据本地实际情况予以少量调整，主要目的是要了解社区居民健康状况和医疗卫生服务利用情况，为本地区改进医疗卫生政策，改善居民健康水平提供信息。所有调查内容仅用于统计分析， 您及家人的信息将予以严格保密。希望您能如实回答下面的问题，非常感谢您的支持与配合！

第一部分：家庭一般情况调查表

表 1 家庭一般情况调查

1	调查前半年内，常住在家里的人数**（包括没有户籍但在您家居住半年以上的人）**？	
2	您家实际生活用房建筑面积约多少平方米? **（建筑面积=使用面积×1.3）**	
3	您家饮水主要类型： 1）自来水 2）桶装水 3）井水 4）其它(请注明 ________________)	
4	您家使用厕所情况： 1）室内厕所 2）室外厕所 3）公共厕所	
5	从您家到最近的医疗点采用适宜方式最快需要多少分钟？	

第二部分 住户成员健康询问调查表

表 2A 住户成员个人基本情况调查

**您家常住人口共有___________人？**		__________ **人**					
**住户成员编码** （01 为户主，其它按调查顺序）		**01**	**02**	**03**	**04**	**05**	**06**
1	住户成员姓名： **（01 填写户主的姓名）**						
2	与户主关系: 1）户主 2）配偶 3）子女 4）孙子/女<br>5）父母 6）祖父/母 7）兄弟姐妹 8）其它（请注明______________）						
3	询问的问题是否将由本人回答： 1)自己回答 2）由他人代答						
4	性别： 1） 男 2） 女						
5	出生年月：□□□□ 年□□月**（如代答者不清楚则不可代答本问卷）**	/	/	/	/	/	/
6	民族：**（填写具体民族名称）** **（如代答者不清楚则不可代答本问卷）**						
7	您目前参加的社会医疗保险是？ （多选）<br>1）公费 2）自费 3）商业保险 4）城镇职工医疗保险<br>5）城镇居民医疗保险<br>6）新型农村合作医疗 7）贫困救助、8）医保（金卡）<br>9）医保（银卡）10）其他						

表 2B 住户成员既往慢性病患病情况调查

**住户成员编码** （01 为户主，其它按调查顺序）	**01**	**02**	**03**	**04**	**05**	**06**

1	您是否患有高血压？ 1）是 2）否 **（答 2 问题 2）**						
1a1	您父母是否患有高血压？1）父亲 2）母亲 3）父母亲都患有 4）父母亲都无						
1a2	您高血压在哪里诊断？**（如该机构类型可归在下面两个以上类别，可选择两个答案）**1）社区卫生服务中心（站） 2）镇医院或二级医院 3）三级综合或专科医院 4）中医医院 5）企事业单位医院/保健站 6) 民营医院 7）其它____						
1a3	您高血压被诊断的时间是哪一年？ □□□□年						
1a4	您高血压是否持续治疗 1）每天吃药 2）间断吃药 3）不舒服才吃药 4）从不吃药						
2	您是否患有糖尿病？ 1）是 2）否 **（答 2 跳问题 3）**						
2b1	您父母是否患有糖尿病？1）父亲 2）母亲 3）父母亲都患有 4）父母亲都无						
2b2	您糖尿病在哪里诊断？**（如该机构类型可归在下面两个以上类别，可选择两个答案）** 1）社区卫生服务中心（站） 2）镇医院或二级医院 3）三级综合或专科医院 4）中医医院 5）企事业单位医院/保健站 6) 民营医院 7）其它____						
2b3	您糖尿病被诊断的时间是哪一年？ □□□□年						
2b4	您糖尿病是否持续治疗？1）每天吃药 2）间断吃药 3）不舒服才吃药 4）从不吃药						
3	您是否患有 COPD(慢性阻塞性肺病)？ 1）是 2）否 **（答 2 跳问题 4）**						
3c1	您 COPD 在哪里诊断？**（如该机构类型可归在下面两个以上类别，可选择两个答案）** 1）社区卫生服务中心（站） 2）镇医院或二级医院 3）三级综合或专科医院 4）中医医院 5）企事业单位医院/保健站 6) 民营医院 7）其它____						
3c2	您 COPD 被诊断的时间是哪一年？ 年						
4	您是否患有脑卒中？1）是 2）否 **（答 2 跳问题 5）**						
4d1	您父母是否患有脑卒中？1）父亲 2）母亲 3）父母亲都患有 4）父母亲都无						

4d2	您脑卒中在哪里诊断？**（如该机构类型可归在下面两个以上类别，可选择两个答案）** 1）社区卫生服务中心（站） 2）镇医院或二级医院 3）三级综合或专科医院 4）中医医院 5）企事业单位医院/保健站 6) 民营医院 7）其它____						
4d3	您脑卒中被诊断的时间是哪一年？ □□□□ 年						
5	您是否患有心脏病？1）是 2）否 **（答 2 跳问题 6）**						
5e1	您心脏病是在哪里诊断？**（如该机构类型可归在下面两个以上类别，可选择两个答案）**1）社区卫生服务中心（站） 2）镇医院或二级医院 3）三级综合或专科医院 4）中医医院 5）企事业单位医院/保健站 6) 民营医院 7）其它____						
5e2	您心脏病被诊断的时间是哪一年？ □□□□ 年						
6	您的眼睛是否存在经医生诊断的疾患？（可多选） 1）无 2）近视 3）远视 4）弱视 5）白内障 6）青光眼 7）糖尿病视网膜病变 8）高血压眼底病变 9）其他						
7	您是否患有经医生诊断的其它慢性疾病?<br>1）是 2）否 **（答 2 跳问表 2C）**						
**如果患有其它慢性病，按就医的经常性依次回答 9 题至 14 题**		-	-	-	-	-	-
8	第一种其它慢性病疾病 （疾病名称）						
9	哪里诊断？**（如该机构类型可归在下面两个以上类别，可选择两个答案）** 1）社区卫生服务中心（站） 2）镇医院或二级医院 3）三级综合或专科医院<br>4)中医医院 5)企事业单位医院/保健站 6) 民营医院 7）其它____						
10	第二种其它慢性病疾病 （疾病名称）**（无则跳问表 2C）**						
11	哪里诊断？**（如该机构类型可归在下面两个以上类别，可选择两个答案）** 1）社区卫生服务中心（站） 2）镇医院或二级医院 3）三级综合或专科医院<br>4)中医医院 5)企事业单位医院/保健站 6) 民营医院 7）其它____						
12	第三种其它慢性病疾病 （疾病名称） **（无则跳问**						

13	哪里诊断？**（如该机构类型可归在下面两个以上类别，可选择两个答案）** 1）社区卫生服务中心（站） 2）镇医院或二级医院 3）三级综合或专科医院<br>4）中医医院 5）企事业单位医院/保健站 6）民营医院 7）其它____						

表 2C 两周患病情况调查（如两周内同一患者患有多种病伤，则每种疾病各填一列，并注明该成员编号，儿童由家长代答）

**住户成员编码** （01 为户主，其它按调查顺序）		**01**	**02**	**03**	**04**	**05**	**06**
1	调查前的 2 周内，是否觉得有身体不适，或患有急、慢性疾病?<br>1）是 2）否 **（答 2 跳问表 2D）**						
2	主要有哪些不适？**（最多选三项）** 1）胸痛 2）腹痛 3）腹泻 4）头痛<br>5）腰腿痛 6）发烧 7）咳嗽 8）心慌/心悸 9）其它_____ 0）无						
3	患的是什么病或受什么伤？ (填疾病名称） 0）无医生明确诊断						
4	所患的病是？1）疾病两周内发生 2）疾病两周前发生延续到两周内						
5	本次病伤在调查前 2 周内持续了多少天？						
6	如您在工作，调查前 2 周内，因本次病伤，休工了多少天？ 99）未工作						
7	如您是学生，调查前 2 周内，因本次病伤，休学了多少天？ 99）未在学						
8	您患病后，这两周内是否进行了治疗（包括自我医疗）？<br>1）是 **（答 1 跳问题 10）** 2）否						
9	未治疗的最主要原因是？**（单选）（答完本题结束，跳问表 2D）**<br>1）自感病轻 2）经济困难 3）无时间<br>4）交通不便 5）医疗服务差 6）自觉无有效措施 7）其它______						
10	如您进行了治疗，采用什么方式？<br>1）纯自我医疗 **（答 1 跳问表 2D）** 2）找医生看病治疗 3）自我治疗并就医						
11	您在哪里看病？**（如在不同的医疗卫生单位看过病，选择次数最多的一个）**<br>1）社区卫生服务中心（站） 2）镇医院或二级医院 3）						

	三级综合或专科医院<br>4）中医医院　5）企事业单位医院/保健站　6) 民营医院　7）其它____						
12	选择上述单位最主要原因是？**（单选）**<br>1）距离近　2）价格低　3）质量好　4）定点单位　5）有熟人　6）有信赖医生　7）服务态度好　8）其它_						
13	看病后，是否根据医生处方在非就诊医院药店配药？<br>1）是　2）否						
14	您最先去的哪里看病？**（如该机构类型可归在下面两个以上类别，可选择两个答案）** 1）社区卫生服务中心（站）2）镇医院或二级医院　3）三级综合或专科医院 4）中医医院　5）企事业单位医院/保健站　6) 民营医院　7）其它____						
15	您此次患病，共花多少医疗费？（包括在医院看病和在院外买药的费用）___元						

表 2D　调查前一年住院治疗情况（如因不同的疾病原因住院，则每种疾病住院情况各填一列，并注明该成员编号，儿童由家长代答）

**住户成员编码**　（01 为户主，其它按调查顺序）		**01**	**02**	**03**	**04**	**05**	**06**
1	在过去的 1 年内，是否因病住过医院?<br>1）是　2）否　**（答 2 跳问表 2E）**						
2	住院原因？1)疾病　2)损伤或中毒　3)康复　4)计划生育　5)分娩　6)其它_____						
3	因疾病或损伤、中毒、康复、计划生育等住院的疾病名称?						
4	调查前一年内, 因这种病伤住过几次医院？						
5	最近一次住院多少天？						
6	最近一次住院的医疗机构类型　**（如该机构类型可归在下面两个以上类别，可选择两个答案）** 1）社区卫生服务中心（站）2）镇医院或二级医院 3）三级综合或专科医院 4)中医医院 5)企事业单位医院/保健站　6) 民营医院　7)其它___						
7	您这次住院，共花了多少医疗费？（包括住院和在院外买药的费用）________元						

表 2E　总体评价情况（儿童由家长代答）

**住户成员编码**　（01 为户主，其它按调查顺序）		**01**	**02**	**03**	**04**	**05**	**06**
1	您生活是否能够自理？1)是 2)否						

2	您是否每天服用 3 种以上处方药？1)是 2)否						
3	您近 3 个月来是否患心理疾患或急性疾病 ？ 1)是 2)否						
4	您活动能力如何？1)卧床或坐椅子 2)能离床或离椅子但不能出门 3)能出门						
5	您是否有神经心理问题？1)严重痴呆或抑郁 2)轻度痴呆 3)无心理问题						
6	您是否有皮肤溃疡? 1)是 2)否						

第三部分 18 岁及以上成年人调查表

表 3A 基本情况

**住户成员编码**（依据第二部分住户成员健康询问调查表中成员编码号）		**01**	**02**	**03**	**04**	**05**	**06**
1	您的婚姻状况: 1）未婚 2）已婚 3）离婚 4）丧偶						
2	您的文化程度:<br>1)文盲 2)小学 3)初中 4)高中技校 5)中专 6)大专 7) 大学及以上						
3	您主要从事的职业: 1）机关事业单位管理者 2）大中型企业高中层管理人员 3）私营企业主 4）专业技术人员 5）办事人员 6）个体工商户 7）商业服务业员工 8) 工人 9) 农民 10) 学生 11) 离退休人员 12）无业人员						

表 3B 健康影响因素

1	您是否吸烟？ 1）不吸烟 **(跳问题 5）** 2）吸烟 3）已戒烟 **(跳问题 4）**						
2	您吸烟多久了？**（不足一年填月份）**						
3	您平均每天吸多少支烟？**(跳问题 5）**						
4	您戒烟多少年了？**（不足一年填月份）**						
5	您平时饮酒吗？<br>1）不饮或很少饮 **（跳问题 8）** 2）偶尔饮 **（跳问题 8）** 3）经常饮						
6	您饮酒多少年了？**（不足一年填月份）**						
7	平均每周饮酒几次?<br>1）每周至少 3 次 2）每周 1-2 次 3）每周不到 1 次						
8	半年来，您业余时间最经常的体育锻炼或健身活动是什						

	么？（**单选**）<br>1）都不参加（**跳问题 11**） 2）走路 3）跑步 4）太极拳类 5）健美操、舞蹈类 6）器械运动 7）球类运动 8）体育比赛 9）其它____________						
9	您平均每周锻炼几次？						
10	平均每次锻炼多少分钟？						
11	11a1 若您不能保证每周参加体育锻炼，最主要原因是什么？<br>1) 从事体力活动，不需要额外运动 2) 没时间锻炼<br>3) 没有适合场所或不方便 4) 身体好，不需要锻炼<br>5） 不愿意活动 6） 身体不好，不能运动<br>7）其它						
	11a2 您每天静坐（包括工作、业余）累计有多少小时？						

表 3C 自我保健情况（此表内容不能代答，代答者跳问表 4A）

1	您知道您的血压值是 1）知道 填收缩压值____ (mmHg） 999）不知道						
2	1）知道 填舒张压值_____(mmHg） 999）不知道						
3	您是否经常主动地获取一些保健知识？ 1)是 2)否						
4	有关卫生保健方面的知识您主要从哪里获得？(**最多选 3 项**）<br>1)医护人员 2)电视 3)广播 4)报刊书籍 5）学校或单位<br>6）同事或亲友 7）墙报 8）网络 9）无 10）其它__________						

表 3D 基本健康知识（此表内容不能代答，代答者跳问表 4A）

**住户成员编码**（依据第二部分住户成员健康询问调查表中成员编码号）		**01**	**02**	**03**	**04**	**05**	**06**
1	过去一年内，您是否接受过社区卫生服务机构的健康教育？<br>1）是 2）否（**答 2 跳问表 3E 题 2**）						
1a1	您参加过的社区卫生服务机构健康教育的形式包括以下哪些（可多选）:<br>1）健康讲座 2） 获得宣传材料 3） 在机构						

	内看过录像　4） 医生面对面的讲授健康知识						
2	您认为健康生活方式主要包括哪些方面：(可多选）<br>1）合理膳食　2） 适量运动　3） 戒烟限酒　4）心理平衡　5） 不知道						
3	您觉得肥胖对健康有什么影响？1）可以增强身体抵抗能力，少生病　2） 没影响　3） 容易引起高血压、糖尿病等许多疾病　4）不知道						
4	您认为被动吸烟对健康有无影响？<br>1）有害　2）无害　3）害处不大　4） 不知道						
5a1	您认为高盐饮食对健康会造成哪些危害？ 易引起传染病 1）是　2）否 9）不知道						
5a2	易引起眼病<br>1）是　2）否 9）不知道						
5a3	易引起高血压 1）是　2）否 9）不知道						
6	您知道一般成年人平均每天吃的食盐量不能超过多少克？_____ **克**　99）不知道						
7a1	您知道什么情况容易患高血压？　肥胖　1）是<br>2）否　9）不知道						
7a2	吃盐多　1）是<br>2）否　9）不知道						
7a3	精神紧张　1）是<br>2）否　9）不知道						
7a4	吸烟　1）是<br>2）否　9）不知道						
7a5	缺乏运动　1）是<br>2）否　9）不知道						
7a6	吃糖多　1）是<br>2）否　9）不知道						
7a7	您认为下面哪个是成年人确诊高血压的诊断标准?（mmHg）<br>1）收缩压≥160 或/和舒张压≥95　2）收缩压≥180 或/和舒张压≥100<br>3)收缩压≥140 或/和舒张压≥90　4)收缩压≥120 或/和舒张压≥80　9）不知道						
7a8	您认为确诊高血压病人是否需要终生药物治疗?<br>1）终生坚持服药　2）血压高时服药　3）有症状时服药　9）不知道						

8b1	您知道什么情况容易患糖尿病？ 肥胖 1）是 2）否 9）不知道						
8b2	吃盐多 1）是 2）否 9）不知道						
8b3	精神紧张 1）是 2）否 9）不知道						
8b4	吸烟 1）是 2）否 9）不知道						
8b5	缺乏运动 1）是 2）否 9）不知道						
8b6	吃糖多 1）是 2）否 9）不知道						
8b7	您知道糖尿病有哪些常见症状吗？						
8b8	吃得多 1）是 2）否 9）不知道						
8b9	喝得多 1）是 2）否 9）不知道						
8b10	尿得多 1）是 2）否 9）不知道						
8b11	体重下降 1）是 2）否 9）不知道						
8b12	吃甜食多 1）是 2）否 9）不知道						
8b13	尿糖多 1）是 2）否 9）不知道						
8b14	您认为确诊糖尿病病人应怎样进行药物治疗?<br>1）终生坚持服药 2）血糖高时服药 3）有症状时服药 9）不知道						
9c1	您知道什么情况容易患慢阻肺？ 吸烟 1）是 2）否 9）不知道						
9c2	饮酒 1）是 2）否 9）不知道						
9c3	接触油烟 1）是 2）否 9）不知道						
9c4	肺炎 1）是 2）否 9）不知道						
10d1	您知道什么情况的人容易患脑卒中吗？高血压 1）是						

	2）否　　9）不知道						
10d2	糖尿病　1）是<br>2）否　　9）不知道						
10d3	高血脂　1）是<br>2）否　　9）不知道						
10d4	酗酒　　1）是<br>2）否　　9）不知道						

表 3E　健康行为知识（此表内容不能代答，代答者跳问表 4A）

**住户成员编码**（依据第二部分住户成员健康询问调查表中成员编码号）		**01**	**02**	**03**	**04**	**05**	**06**
1	您多长时间进行一次健康检查？<br>1）1 次/半年　2）1 次/1 年　3）1 次/2 年　4）不定期　5）从不参加体检						
2	您是否每天都有吃蔬菜?　1）是　　　　2）否						
3	您是否每天都有吃水果?　1）是　　　　2）否						

表 3F　社区卫生服务站知晓与利用（此表内容不能代答，代答者跳问表 4A）

**住户成员编码**（依据第二部分住户成员健康询问调查表中成员编码号）		**01**		**02**		**03**		**04**		**05**		**06**	
1	最近一年里，您平时就诊最多的医疗机构是？**(选 1 个)**<br>1）社区卫生服务中心（站）　2）镇医院或二级医院　3）三级综合或专科医院<br>4）中医医院　5）企事业单位医院/保健站　6) 民营医院　7）其它____<br>**(答 1 者跳问题 4)**												
2	您知道附近有社区卫生服务中心（站）吗？ 1）知道　2）不知道(**答 2 跳问表 4A**）												
3	您去过该社区卫生服务中心（站）吗？ 1）去过　2）没去过(**答2 跳问表4A**）												
4	您步行到该站或中心在路上需花多少分钟？99）不知道												
5	您到那里去的目的是？(**可多选，最多选 4 项**）<br>1）看病　2）开药　3）慢性病随访　4）咨询　5）针灸、理疗　6）预防接种　7）儿童查体　8）孕妇查体　9）健康体检　10）其它_______												

第四部分　特殊人群调查表

表 4A　60 岁及以上老年人情况调查

**住户成员编码**（依据第二部分住户成员健康询问调查表中成员编码号）		01	02	03	04	05	06
1	正在进行的调查对象年龄在 60 岁及以上吗？ 1）是　2）否 **(答 2 跳问表 4B)**						
2	您的食欲（或胃口）如何？1）很好　2）好　3）一般　4）差　5）很差						
3	您的睡眠如何？　1）很好　2）好　3）一般　4）差　5）很差						
4	您的日常生活是否需要依靠药物或医疗帮助？<br>1）根本不需要　2）偶尔需要　3）一般需要　4）多数需要　5）完全需要						
5	总的来讲，您感觉自己的健康状况如何？<br>1）很好　2）好　3）一般　4）差　5）很差						
6a	近一年来，您做过健康体检吗？　1）在医院做过　2）在体检机构做过　3）在社区卫生服务机构做过　4)没做过　9）不知道						
6b	近一年来，社区卫生服务机构的医务人员主动与您联系过几次？____次<br>0）没联系过 99）记不清						
7. **(最近 1 个月）您从事下列活动有无困难？程度如何？**		-	-	-	-	-	-
7.1	骑单车或做家务 1 个小时以上：1）毫无问题　2）有点困难　3）比较困难　5）完全不能						
7.2	步行上三层楼：　1）毫无问题　2）有点困难　3）比较困难　5）完全不能						
7.3	弯腰或曲膝、下蹲：<br>1）毫无问题　2）有点困难　3）比较困难　5）完全不能						
7.4	步行 1~2 里路：<br>1）毫无问题　2）有点困难　3）比较困难　5）完全不能						
8. **(最近 1 个月）您的心理状态如何？**		-	-	-	-	-	-
8.1	您是否感觉生活充实和快乐？<br>1）总是有　2）经常有　3）时有时无　4）偶尔有　5）完全没有						
8.2	您有情绪低落或忧郁等情况吗						

	1）完全没有 2）偶尔有 3）时有时无 4）经常有 5）总是有						
8.3	您的记忆力怎样？ 1）很好 2）好 3）一般 4）差 5）很差						
8.4	您能够专注地做一件事持续 10 分钟以上吗？ 1）完全能 2）多数能 3）一般能 4）偶尔能 5）根本不能						
9.	**（最近 1 个月）您的社会关系情况**	-	-	-	-	-	-
9.1	您的家庭关系如何？ 1）很好 2）好 3）一般 4）差 5）很差 6）无来往						
9.2	您的亲友关系如何？ 1）很好 2）好 3）一般 4）差 5）很差 6）无来往						
9.3	您的邻居关系如何？ 1）很好 2）好 3）一般 4）差 5）很差 6）无来往						

表 4B 50 岁以下已婚妇女情况调查

**住户成员编码**（依据第二部分住户成员健康询问调查表中成员编码号）		**01**	**02**	**03**	**04**	**05**	**06**
1	您是否患过妇科疾病?（可多选） 1）无 2）阴道炎 3）宫颈炎 4）宫外孕 5）附件炎 6）卵巢肿瘤 7）子宫肌瘤 8）其它						
2	您近两年来是否接受过妇女病普查？ 1）是 2）否 9）不记得						
3	您近一年来是否经医生做过乳腺检查？ 1）是 2）否 9）不记得						
4	您知道乳腺自查的方法吗？ 1）知道 2）不知道						
5	过去一年里您是否做过人工流产？ 1）是 2）否						

表 4C 0-6 岁以下儿童情况调查（儿童家长代答）

**住户成员编码**（依据第二部分住户成员健康询问调查表中成员编码号）		**01**	**02**	**03**	**04**	**05**	**06**
1	您和孩子的关系是？ 1）母亲 2）父亲 3）祖父母/外祖父母<br>4）家庭其他成员（请注明）____ 5）其他人（请注明）______						
2	您的孩子是否参加系统儿童保健管理？						

	1）是 2）否 9）不清楚(**答 2 或 9 跳问题 4**）						
3	您的孩子在哪里进行系统保健管理？（如该机构类型可归在下面两个以上类别，可选择两个答案） 1）社区卫生服务站 2）医院 3）企事业职业医院/保健站 4）预防保健机构 5）其它____________						
4	您知道婴儿应该添加辅食的月龄是？（辅食指谷类或米粉类泥糊状食物）<br>___月 99）不知道						
4a	您认为在给孩子添加辅食之前，至少要对他（她）进行几个月的纯母乳喂养？<br>___月 99）不知道						
4b	您的孩子大约几个月的时候断的奶？____个月<br>0）从未吃母乳 88) 正在吃奶 99）不知道						
5	您认为孩子学爬重要吗？1）必须学爬 2）应该学爬 3） 不需要学爬 9） 不知道						
6	您的孩子有下列哪些饮食习惯与行为？（**可多选**）<br>1）无不良饮食习惯<br>2）挑食、偏食 3）多零食 4）贪食 5）边吃边玩或看电视						
7	孩子的口腔护理						
7a1	3 岁以下儿童：<br>1）吃奶后喂白开水 2）软布或手指刷清洁 3) 软牙刷刷牙 4）没有						
7a2	3 岁以上儿童：几岁开始刷牙？___岁 0）现在还没有开始刷牙 99）不记得						
8	您的孩子每日户外平均活动几小时？(填小时数___)<br>99）不清楚						
9	您的孩子平均每日看电视累计几小时？ (填小时数___)<br>99）不清楚						
10	未经医生允许您曾经常给孩子吃保健品吗？ 1）经常 2）偶尔 0）从未吃过						
11	未经医生允许您曾经常给孩子吃 “中、西药”吗？<br>1）经常 2）偶尔 0）从未吃过						
11a	您知道给孩子补铁有什么好处吗？<br>1）预防肥胖 2）预防佝偻病 3）预防贫血 4）不知道						
11b	您知道下列哪种食物可以为孩子补铁吗？<br>1）动物肝脏 2）米粥 3）牛奶 4）不知道						
11c	孩子六个月后，医生建议过您给孩子多吃动物肝脏或瘦						

	肉吗？<br>1）医生建议过　2）医生没建议过　3）孩子没到六个月　9）不清楚						
11d	您或您的家人会在孩子面前吸烟吗？<br>1）经常　2）偶尔　3）从不　4）家里无人吸烟						
12	您的孩子多大进保育院或幼儿园？**（3 岁以下按月龄计算）**___个月<br>**（3 岁以上按年龄计算）**___岁　0）未进						
13	您的孩子曾有过意外伤害吗？　1）有过　2）无**（跳到 14a）**						
13a1	最近一次意外伤害是在哪一年？　______年　伤害的原因是：<br>1）摔伤　2）撞伤　3）利器伤　4）烫伤　5）烧伤　6）中毒　7）其它____						
14a	从孩子出生至今，打预防针的日期临近时有没有医务人员提醒过您？　1）总是有人提醒　2）偶尔提醒过　3）从来没人提醒　4）不知道						
14b	从孩子出生至今，当体检日期临近时有没有医务人员提醒过您？　1）总是提醒　2）偶尔提醒　3）从来没有提醒　4）不知道						
15a	在过去的三个月中，您用于给孩子体检和看病的花费是多少？<br>1）<100　2）100-300　3）300-500　4）500-1000　5）>1000 元						

表 4D　7～17 岁学龄期未成年人情况调查（自答）

**住户成员编码**（依据第二部分住户成员健康询问调查表中成员编码号）		**01**	**02**	**03**	**04**	**05**	**06**
1	您每天吃早餐？　1）是　2) 否						
2	您有下列哪些饮食习惯与行为？**（可多选）**　1）无不良饮食习惯　2）挑食、偏食　3）多零食　4）贪食　5）边吃边玩或看电视						
3	您每天刷牙嗎？　1）是　2) 否						
4	您平均每日（包括假日）看电视和接触电脑几小时？(填小時數___)<br>99）不清楚						
5	您觉得睡眠好吗？　1）好　2）一般　3）不好<br>9）不清楚						

7	您有过下列表现吗?**（可多选，最多选 3 个）**<br>1）多动 2）抽动 3）强迫 4）攻击 5）交往障碍 6）学习障碍<br>7）退缩 8）自闭倾向 9）不清楚 0）无上述表现						
8	您与同学关系好吗？ 1）很好 2）一般 3）不好 9）不清楚						
9	您遇到挫折或情绪波动愿意与家长倾诉或沟通吗？<br>1）愿意沟通 2）不问不说 3）问也不说 9）不清楚						
10	您知道男孩和女孩的青春期标志吗？ 1）回答正确 2）回答错误 9）不清楚						
11	您有早恋的异性朋友吗？ 1）有 2）没有 9）不清楚						

表4E　孕产妇情况调查表

**住户成员编码**（依据第二部分住户成员健康询问调查表中成员编码号）		**01**	**02**	**03**	**04**	**05**	**06**
1a	正在进行的调查对象是正在怀孕或 1 岁内孩子的妈妈吗？<br>1）正在怀孕 **(初孕者答 1 后不需答题 6)** 2）1 岁内孩子的妈妈 3）都不是 **(答 3 跳问表 Q）**						
2a	本次怀孕之前，您是否出现过以下情况？<br>1）流产 2）习惯性流产 3）死胎 4）死产 5）畸形儿 6）智力低下儿 7）都没有						
3a	您这次怀孕是计划好的吗？ 1）是 2）否 **（答 2 跳至题 4a）**						
3b	在您准备怀孕之前，医生是否建议您进行孕前的健康检查？<br>1）没有得到任何建议 2）社区医生建议过 3）妇产科医生建议过						
3c	您和丈夫是否接受过专门的孕前健康检查？<br>1）都没有 2）本人检查过 3）丈夫检查过 4）夫妻双方均检查过 5) 不知道						
4a	您知道孕前补充叶酸的作用是什么？<br>1）预防母亲高血压 2）预防胎儿兔唇 3）预防胎儿神经管畸形 9）不知道						
4b	您什么时候开始补充的叶酸？ 1）孕前一年 2）孕前三个月 3）发现怀孕时 4）孕后三个月 5）没补过 9）不清楚						

5a	您在孕______周时建立孕产妇保健手册？ 99）未建册						
5b	您怀孕期间是否接受过孕期保健服务？ 1）是 2）否 **（答 2 跳至题 6a）**						
5c	您在哪个医疗机构做的孕期保健（如该机构类型可归在下面两个以上类别，可选两个答案） 1）社区卫生服务机构 2）区妇幼保健院 3）市妇幼保健院 4）二级医院（综合性医院） 5）三级医院（综合性医院） 6）私立医院						
6a	您生完孩子出院后多长时间社区医务人员第一次到您家上门访视?<br>1)1 周以内 2) 第 2 周 3) 第 3 周及以上 4)没有医务人员来访视 **（答 4 跳至表 Q）**						
6b	社区医务人员到您家访视一共几次？ ___ 次 9）不知道						

第五部分 体格检查（18 岁及以上人群）

**住户成员编号**（依据第二部分住户成员健康询问调查表中成员编码号）		**01**	**02**	**03**	**04**	**05**	**06**
1	您今天服过降压药、扩血管药、利尿药、镇静类药物吗?<br>1）是 2）否						
2	过去半小时内，您是否吸烟、饮酒/咖啡或进行过剧烈运动？ 1）是 2）否						
3	第一次测量：左侧血压： 收缩压（mmHg）/舒张压（mmHg）	/	/	/	/	/	/
4	右侧血压： 收缩压（mmHg）/舒张压（mmHg）	/	/	/	/	/	/
	**休息 1 分钟后测量第二次血压**						
5	第二次测量：左侧血压： 收缩压（mmHg）/舒张压（mmHg）	/	/	/	/	/	/
6	右侧血压 收缩压（mmHg）/舒	/	/	/	/	/	/

	张压（mmHg）						
7	身高 （厘米）（保留 1 位小数）						
8	体重 （千克）（保留 1 位小数）						
9	腰围 （厘米）（保留 1 位小数）						
10	臀围 （厘米）（保留 1 位小数）						
11	上臂肌围（厘米）（保留 1 位小数）						
12	小腿周径（厘米）（保留 1 位小数）						
13	血糖值						
